# Seasonality influences skin bacterial community structure and anti-Bd function in two anuran species

**DOI:** 10.3389/fmicb.2024.1463563

**Published:** 2024-11-05

**Authors:** Han Zhang, Hongying Ma, Jie Deng, Hu Zhao, Cheng Fang, Jianlu Zhang, Qijun Wang, Hongxing Zhang, Wei Jiang, Fei Kong

**Affiliations:** Shaanxi Key Laboratory of Qinling Ecological Security, Shaanxi Institute of Zoology, Xi’an, China

**Keywords:** seasonal variation, host species difference, skin, bacterial community, anurans, amphibians

## Abstract

Microorganisms on amphibian skin reduce disease susceptibility and play an important role in pathogen defense. We hypothesized that anuran skin bacterial communities would change in response to seasonal variation and host species. To test this hypothesis, we used 16S rRNA amplicon sequencing to identify cutaneous bacterial communities of two frogs from the Qinling Mountains of China, *Pelophylax nigromaculatus* and *Nanorana quadranus*. We matched the amplicon sequence variants (ASVs) of microbes exhibiting protective effects against the pathogen *Batrachochytrium dendrobatidis* (Bd), using a database containing over 1900 16S rRNA gene sequences from amphibian skin bacteria. The results showed that seasonal variation had a stronger effect than host species on the structure (alpha-diversity, beta-diversity, species composition and abundance, and biomarkers) and anti-Bd function of cutaneous bacterial communities. These communities were highly dynamic but varied similarly between hosts. Their structural similarities were more consistent at the phylum level, but markedly less so at finer taxonomic levels. The highest relative abundance of anti-Bd reads was observed in *P. nigromaculatus* during summer, but anti-Bd reads were present in both frog species during different seasons. Therefore, the protective function of cutaneous microbial communities appears to be continuous despite between-species differences in anti-Bd ASV abundance. This observation does not directly explain why Bd infections have not been recorded in the region, butprovides important insight on anuran pathogen defense mechanisms. Our findings also suggest that specific seasons can be periods of high infection risk, with major implications for research on amphibian ecology and conservation.

## 1 Introduction

Bacterial communities on the skin are an important first line of defense against pathogens in amphibians (Brunetti et al., 2021; Jani et al., 2021; Longo et al., 2015). Experimentally removing skin bacteria increases morbidity associated with chytridiomycosis, whereas the addition of certain nascent bacteria reduces morbidity and mortality in some amphibian species (Becker et al., 2009; Harris et al., 2009; Kueneman et al., 2016; Walke et al., 2015). Symbiotic bacterial communities have gained greater recognition in mediating protection against a wide range of pathogens by modulating and contributing to host immunity (Bletz et al., 2017b; Fraune et al., 2015; Khosravi and Mazmanian, 2013; Rosenberg et al., 2007; Woodhams et al., 2007). Moreover, skin bacterial protection may be linked to specific bacterial metabolites and volatile compounds (Becker et al., 2009; Woodhams et al., 2018). For instance, violacein, prodigiosin, and volatile organic compounds produced by amphibian skin bacteria inhibit two species of chytrid fungi, *Batrachochytrium dendrobatidis* (Bd) and *B. salamandrivorans* (Bsal) (Brucker et al., 2008a; Woodhams et al., 2018), the criminal ringleader of amphibian chytridiomycosis.

Chytridiomycosis is a fatal skin disease that has significantly contributed to global decline and extinction of amphibian populations (Scheele et al., 2019). Of the two pathogens, Bd exhibits a broader host range, including a large number of Anuran, Caudate, and Gymnophiona species. While Bsal typically infects salamanders, it can also affect anuran species, including *Osteopilus septentrionalis* (Basanta et al., 2022; Gray et al., 2023; Laking et al., 2017; Towe et al., 2021). Bd infection exhibits temporal variation, and Bsal infection may exhibit similar pattern (Bletz et al., 2017b; Longo et al., 2015; Kinney et al., 2011; Longo et al., 2010; Phillott et al., 2013). This temporal variation is largely driven by temperature fluctuations (Bletz et al., 2017b). Therefore, temporal or seasonal variations in skin microbiota may be an important factor in disease dynamics. Amphibian skin microbial communities may also vary in response to habitat and pathogen presence, as well as host-specific factors such as species and developmental stage (Bletz et al., 2017a; Longo and Zamudio, 2017; McKenzie et al., 2012; Prest et al., 2018; Rebollar et al., 2016; Rebollar et al., 2019).

The Qinling Mountains serve as the natural demarcation line between northern and southern China. These mountains are important biodiversity hotspots but have recently experienced a sharp decline in amphibian populations. The most evident causes include climate change and habitat destruction, but pathogen infection has not been ruled out. Interestingly, no Bd- or Bsal-infected amphibians have been reported to date. However, no studies have investigated the assemblages and structures of amphibian cutaneous bacterial community in the Qinling Mountains; hence, we cannot conclude whether the lack of infection is due to superior anti-pathogen protection on amphibian skin or an absence of pathogens in the region.

To address these knowledge gaps, our study focused on two questions. First, what happens to the composition and diversity of amphibian cutaneous bacteria communities in response to seasonal variation and host species differences? Second, do any resultant fluctuations in microbial communities influence their protective function against pathogens such as Bd? We obtained data from two common and abundant aquatic anuran species in the Qinling Mountains, *Pelophylax nigromaculatus* and *Nanorana quadranus*. We sampled the cutaneous bacterial community across seasons, calculated their diversity and structure, as well as assessed whether chytridiomycosis was present and amplicon sequence variants (ASVs) from cutaneous microbes exhibited anti-Bd properties. Our findings should facilitate the elucidation of the cutaneous microbial ecology of frogs from the Qinling Mountains. Additionally, understanding the biological mechanism associated with pathogen susceptibility, even in species that are not currently in decline, can help us to establish how this range of susceptibility relates to the cutaneous skin microbiota of amphibians.

## 2 Materials and methods

### 2.1 Sample collection

Adult individuals from two aquatic anurans [the running-water frog, *N. quadranus* (*n* = 20), and the quiet-water frog, *P. nigromaculatus* (*n* = 12)] were sampled in spring, summer, and autumn of 2023 at Huangbaiyuan, located in the southern foot of the Qinling Mountains. Frogs were not sampled in winter because they hibernate during that season. The two species were selected for their abundance, increasing the likelihood of obtaining adequate sample sizes across seasons. For details on specific sampling times and locations (see Table 1).

**Table 1:** Sample information of anuran species included in this study.

Species	Sampling time	Sample size	Location	Elevation (m)	Site description
*Pelophylax nigromaculatus*	25th, April	8 (F, 4; M, 4)	33.70964668°N 107.39150169°E	989	Paddy fields
	17th, July	6 (F, 3; M, 3)			
	19th, September	6 (F, 2; M, 4)			
*Nanorana quadranus*	25th, April	\	33.70561351°N 107.38986328°E	988	Stream
	17th, July	6 (F, 3; M, 3)			
	19th, September	6 (F, 3; M, 3)			

Frogs were captured manually by researchers wearing sterile high-density polyethylene gloves (one pair per subject) and then rinsed thrice with purified water to flush away dirt and transient bacteria. Skin on the back, abdomen, and limbs was swabbed 30 times using a sterile skin swab. These swabs were placed in 2 mL of DNA storage solution (consisted of Tris, EDTA-2Na, and NaCl; Shanghai Langfu Industrial) and stored at 4°C. Collected samples were transported on dry ice to Beijing Biomarker Technologies for sequencing.

### 2.2 DNA extraction and sequencing

Total genomic DNA was extracted from all swabs using a TGuide S96 Magnetic Soil/Stool DNA Kit (Tiangen Biotech, Beijing, China), including lysozyme pretreatment. Extracted DNA was used as templates to amplify the V4 region of the 16S rRNA gene using barcoded primers pairs (515F: 5′-GTGYCAGCMGCCGCGGTAA-3′; 806R: 5′-GGACTACNVGGGTWTCTAAT-3′) in a polymerase chain reaction (PCR) reaction (Bletz et al., 2017b). The PCR products were quantified through agarose gel electrophoresis and purified using a DNA purification kit (Omega, Norcross, GA, USA). Purified amplicons were subjected to paired-end sequencing (2 × 250 bp) on the Illumina Novaseq 6,000 platform.

### 2.3 Sequence processing

Sequence processing and analyses were performed using BMK Cloud.^1^ Raw data were primarily filtered based on single-nucleotide quality in Trimmomatic (version 0.33) (Bolger et al., 2014). Primer sequences were identified and removed in Cutadapt (version 1.9.1) (Martin, 2011).

Further quality control of data was performed using filterAndTrim function, setting maxEE to 2 [EE = sum(10^(−Q/10))] and other parameters as default. Model construction was performed using learnErrors function, de-noising was performed with dada2 function, and double-end reads splicing was performed using mergePairs function (setting parameters: minOverlap: 18, maxMismatch: 18*0.2). Chimera removal was performed using the removeBimeraDenovo function (select the consensus method) (Edgar et al., 2011; Edgar, 2013; Callahan et al., 2016).

### 2.4 Sequence analysis

Feature classification was conducted using clean reads to generate ASVs through dada2, and ASVs with counts <2 across all samples were filtered (Callahan et al., 2016). These ASVs were matched against the SILVA database (release 138.1) in QIIME2 for taxonomic annotation, based on the Naive Bayes classifier with a confidence threshold of 70% (Quast et al., 2013).

Shannon, Simpson, Chao1 and ACE indices were calculated for frog skin samples using QIIME2 and displayed using ggplot2 (version 3.1.1). Between-group (species) differences in alpha diversity were determined with Wilcoxon test. Significance was set at *p* < 0.05. Beta diversity was calculated with unweighted UniFrac and Binary jaccard and visualized with principal coordinates analysis based on dissimilarity matrices (PCoA) (Bolyen et al., 2019). Between-group differences in beta diversity were tested using PERMANOVA [Adonis function in the R (version3.1.1) Vegan (version 2.3–0) package] (Dixon, 2003; Anderson, 2017). Unweighted pair group method with arithmetic mean (UPGMA) in Python was used to determine clustering patterns across samples. Linear discriminant analysis-effect size (LEfSe) was used to test for significant taxonomic differences between groups (Segata et al., 2011). A logarithmic LDA score of 4.5 was set as the threshold for discriminative features to analyse the effect of season on bacterial communities, while the LDA score of 4.0 was set to analyse the effect of host species on bacterial communities.

Next, to explore the protective effect of cutaneous bacterial communities, a database was utilized which containing over 1900 16S rRNA gene sequences from amphibian skin bacteria that have been tested for activity against the pathogen, Bd (Woodhams et al., 2015). Positive hits were then matched with ASVs present in the samples to calculate the proportion of anti-Bd reads (100%matching rate). Correlations between proportion of inhibitory reads and seasonal or species differences were tested using ANOSIM.^2^

In the sequence analysis, frog samples were divided into groups based on capture date. Therefore, *P. nigromaculatus* caught in spring, summer, and autumn were, respectively, labeled as “SpringPn” (SpringPn1–8), “SummerPn” (SummerPn1–6), and “AutumnPn” (AutumnPn1–6). Because no *N. quadranus* was caught in the spring, these frogs were divided into two groups: “SummerNq” (SummerNq1–6) and “AutumnNq” (AutumnNq1–6) (Table 2).

**Table 2:** The number of amplicon sequence variants (ASVs) and alpha diversity indices in each sample.

Group	Sample	ASV number	Abundance index		Diversity index	
			Chao1	ACE	Shannon	Simpson
SpringPn	SpringPn1	406	406.5	406.4364	5.3235	0.8584
	SpringPn2	451	451.6	451.6352	5.5888	0.8912
	SpringPn3	368	369.1111	368.9486	5.2654	0.8988
	SpringPn4	379	379.0769	379.4055	4.8156	0.8623
	SpringPn5	533	538.625	535.0931	5.7751	0.8969
	SpringPn6	475	475.1111	475.3453	5.7726	0.8741
	SpringPn7	422	422.0909	422.3238	4.8648	0.8453
	SpringPn8	458	458.7895	459.3034	4.4526	0.8191
SummerPn	SummerPn1	266	267.5	266.8661	5.7775	0.9522
	SummerPn2	289	290.75	291.1224	4.8987	0.9011
	SummerPn3	195	199.375	201.1858	2.2206	0.581
	SummerPn4	264	265.5	266.0122	3.7751	0.7001
	SummerPn5	260	262.0	260.8191	5.9919	0.9684
	SummerPn6	256	256.0	256.1788	5.9082	0.9603
AutumnPn	AutumnPn1	750	750.7143	750.8289	6.8989	0.9323
	AutumnPn2	896	896.5	896.6	8.5164	0.9852
	AutumnPn3	903	904.9091	904.0607	8.0817	0.9759
	AutumnPn4	892	893.0	892.751	8.0666	0.9716
	AutumnPn5	899	899.75	899.4297	8.9083	0.9953
	AutumnPn6	928	928.8333	928.8036	9.069	0.9964
SummerNq	SummerNq1	299	300.5	300.3972	5.9934	0.9663
	SummerNq2	249	249.0	249.2215	5.5122	0.9443
	SummerNq3	258	264.875	261.0846	3.138	0.5883
	SummerNq4	356	358.0	357.0349	5.9816	0.9418
	SummerNq5	282	282.25	282.4167	6.0471	0.9544
	SummerNq6	274	275.5	274.5855	5.9061	0.9613
AutumnNq	AutumnNq1	892	892.8571	892.7027	9.0916	0.9964
	AutumnNq2	950	951.25	950.8267	9.2332	0.9971
	AutumnNq3	868	869.875	869.0421	9.0158	0.9962
	AutumnNq4	769	770.0714	770.1384	8.5982	0.994
	AutumnNq5	898	899.1111	898.8503	8.8625	0.9945
	AutumnNq6	757	761.5	758.6142	8.2892	0.9865

### 2.5 Detection of chytridiomycosis

The extracted DNA was then subjected to detection through PCR utilizing Bd and Bsal nested primer pairs. The outer nest primer pairs of Bd and Bsal are ITS1f1 (5′- CTT GGT CAT TTA GAG GAA GTAA −3′) and ITS4 (5′-TCC TCC GCT TAT TGA TAT GC-3′). The inner nest primer pairs of Bd are Bd1a (5’-CAGTGTGCCATATGTCACG-3′) and Bd2a (5’-CATGGTTCATATCTGTCCAG-3′), and the inner nest primer pairs of Bsal are STerF (5′TGCTCCATCTCCCCCTCTTCA3′) and STerR (5′TGAACGCACATTGCACTCTAC3′).

The PCR reaction system consisted of 2 × Taq PCR Mix II (catalog# KT211-02, Tiangen Biotech) in a volume of 10 μL, with 1 μL each of upstream and downstream primers (10 μM), 1 μL of template DNA, and 7 μL of deionized water (ddH_2_O), resulting in a total reaction volume of 20 μL. The PCR amplification conditions were 10 min at 95°C, followed by 30 cycles of 10 s at 95°C, 10 s at 53°C (outer nest primer pairs) /52°C (inner nest primer pairs of Bd) /58°C (inner nest primer pairs of Bsal), and 10 s at 72°C and a final 10 min at 72°C. The resulting target gene fragments of Bd and Bsal were of lengths 296 bp and 161 bp, repectively.

## 3 Results

### 3.1 Overview of cutaneous bacterial communities

After sequencing, data filtering, and sequence splicing of 16S rRNA amplicons from 33 frog skin samples, we obtained 1,054,720 sequences. These sequences were further processed and clustered into 1943 ASVs, predominantly from five phyla: Proteobacteria (558), Firmicutes (283), Actinobacteriota (199), Bacteroidetes (276), and Acidobacteriota (157).

**Figure 1:**
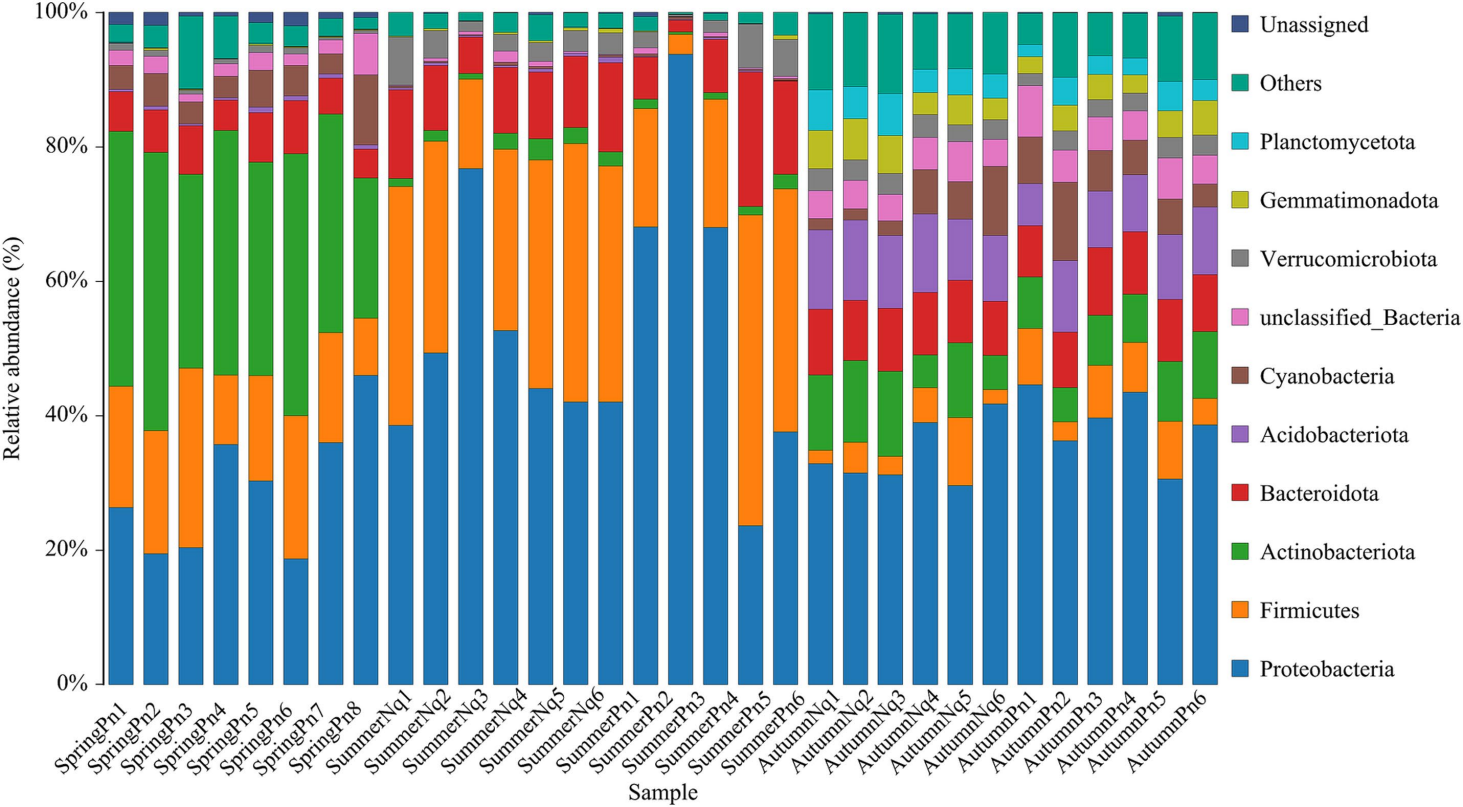
Histogram of skin microbial distribution at the phylum level. Different colors indicate different species; stacked columns are the top 10 taxa in relative abundance at each taxonomic level.

Table 2 and Figure 1 show the number of ASVs per sample and relative ASV abundance by phyla per sample, respectively. We found 88 ASVs common to all samples, belonging to Proteobacteria (38), Firmicutes (17), Bacteroidota (15), Actinobacteriota (10), Acidobacteriota (2), Cyanobacteria (1), Acidobacteriota (1), Verrucomicrobiota (1), Desulfobacteriota (1), Fusobacteriota (1), unclassified_Bacteria (1) and Unassigned (1) (Supplementary Figure 1).

### 3.2 Seasonal variation influenced bacterial communities

The SpringPn, SummerPn, and AutumnPn groups shared 202 ASVs, or 10.66% of total ASVs found in these samples (Supplementary Figure 2). The SummerNq and AutumnNq groups shared 255 ASVs, accounting for 14.74% of the total across both datasets (Supplementary Figure 3).

**Figure 2:**
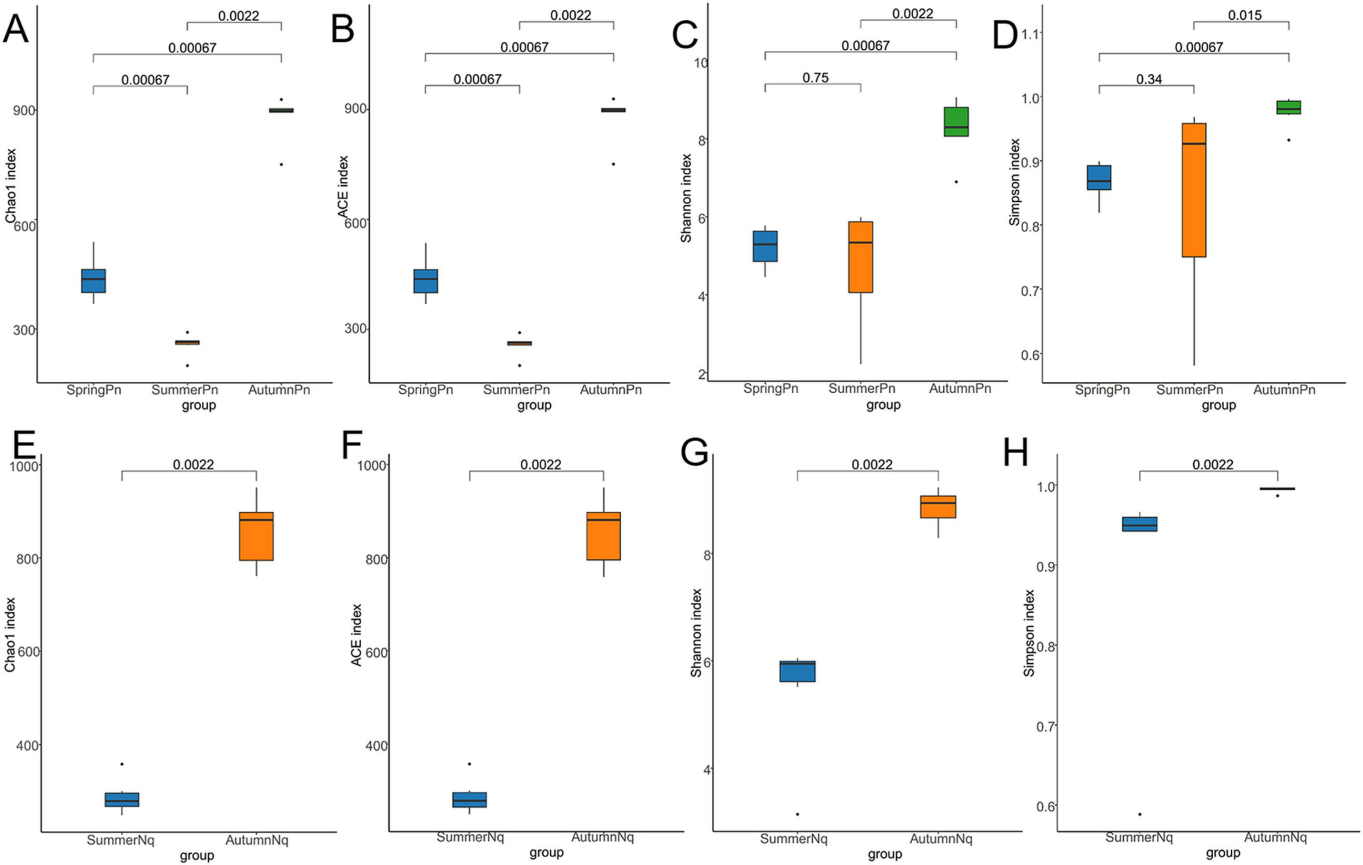
Box plot of variation in alpha diversity indices for cutaneous bacteria on *Pelophylax nigromaculatus* across three seasons (A–D) and on *Nanorana quadranus* across two seasons (E–H). The horizontal coordinates are the group names, and the vertical coordinates are the values of the corresponding alpha diversity indices. (A,E) Wilcoxon test of Chao1 index; (B,F) Wilcoxon test of ACE index; (C,G) Wilcoxon test of Shannon index; (D,H) Wilcoxon test of Simpson index.

The Chao1 and ACE indices showed that the microbial species richness of *P. nigromaculatus* significantly differed across the three seasons, decreasing in the order of AutumnPn > SpringPn > SummerPn (Table 2; Figures 2A,B). Shannon and Simpson indices showed that the AutumnPn group had significantly greater species diversity than the SpringPn and SummerPn groups; however, the latter two groups did not differ (*p* > 0.05, Table 2; Figures 2C,D). For *N. quadranus*, species richness and diversity were significantly greater in the AutumnNq group than in the SummerNq group (Figures 2E–H).

**Figure 3:**
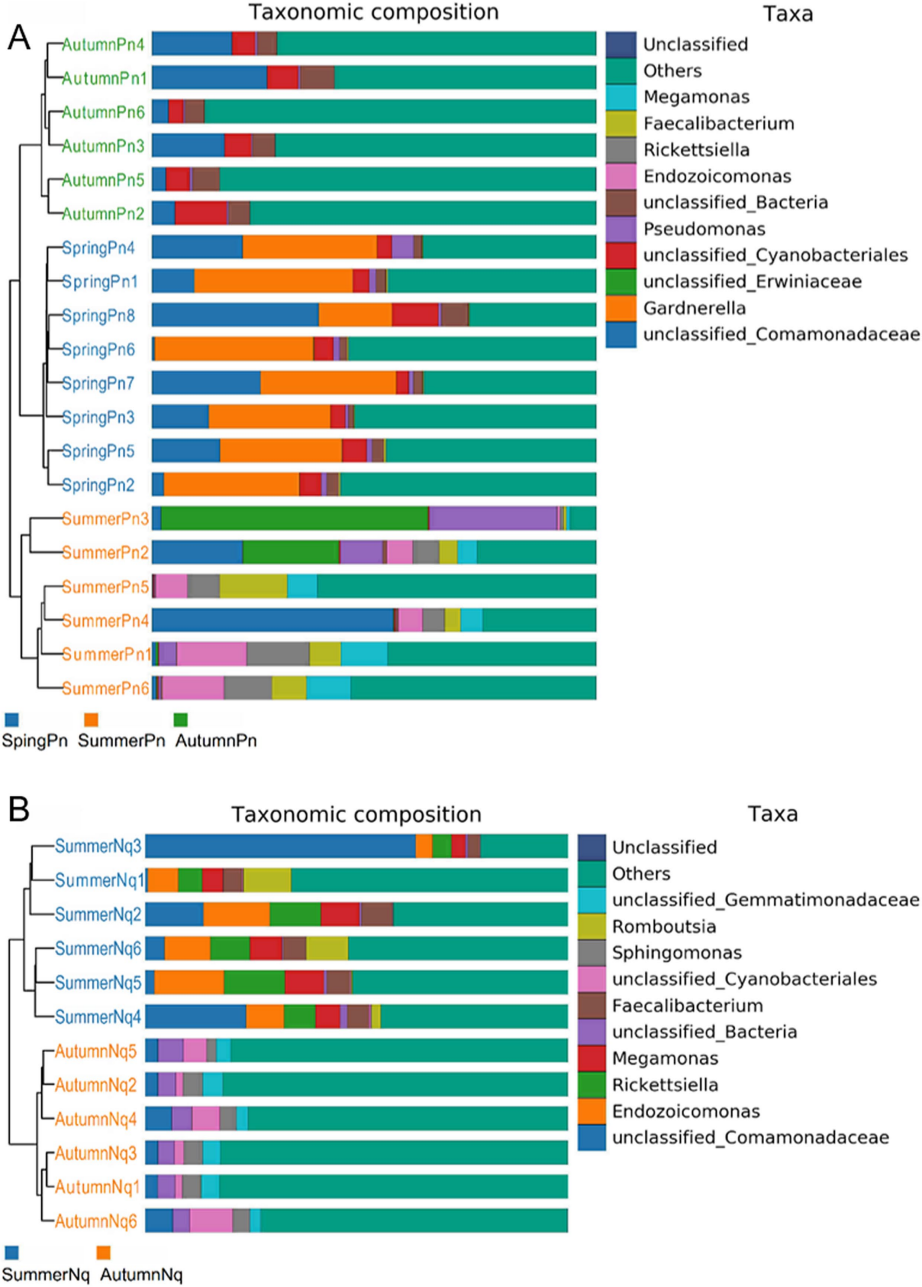
Principal coordinates analysis of beta diversity in cutaneous bacteria on all groups. (A) unweighted UniFrac distance; (B) Binary jaccard distance. Each point represents the skin bacterial community of an individual sample.

Using PCoA to analyse the unweighted UniFrac and Binary jaccard distance indices, we found seasonal differences in the species composition of cutaneous bacterial communities. On the PC1 axis, SummerPn clustered to the left, AutumnPn to the right, and SpringPn in the center. A large gap in their bacterial communities existed between the three seasons. On the PC2 axis, SpringPn clustered on the upper side, while SummerPn and AutumnPn were separated by a large gap with SpringPn (Figures 3A,B). For *N. quadranus*, the PC1 axis was the main factor contributing to a large difference between SummerNq and AutumnNq (Figures 3A,B). PERMANOVA (*p* = 0.001, Treatments 1, 2 in Tables 3, 4) verified the seasonal difference in the bacterial communities between the different seasons for both frog species.

**Table 3:** Summary of PERMANOVA models (unweighted UniFrac distance) of beta diversity for microbial communities on frog skin.

Treat	Variables	Degrees of freedom	Sums of squares	Mean squares	F. model	*R* ^2^	*P*
1	*P. nigromaculatus* in different seasons	2	2.205844	1.102922	20.11499	0.702953	0.001**
2	*N. quadranus* in different seasons	1	1.428132	1.428132	20.33018	0.670295	0.001**
3	*P. nigromaculatus* and *N. quadranus* in summer	1	0.098588	0.098588	0.911988	0.083577	0.309
4	*P. nigromaculatus* and *N. quadranus* in autumn	1	0.030326	0.030326	0.868604	0.079919	0.459

Effects on variation due season (treat 1 and treat 2), and species (treat 3 and treat 4) are considered. Significant results are marked with an asterisk.

**Table 4:** Summary of PERMANOVA models (Binary jaccard distance) of beta diversity for microbial communities on frog skin.

Treat	Variables	Degrees of freedom	Sums of squares	Mean squares	F. model	*R* ^2^	*P*
1	*P. nigromaculatus* in different seasons	2	3.606148	1.803074	11.90274	0.583389	0.001**
2	*N. quadranus* in different seasons	1	2.010845	2.010845	13.16891	0.568387	0.001**
3	*P. nigromaculatus* and *N. quadranus* in summer	1	0.225315	0.225315	1.068052	0.096499	0.212
4	*P. nigromaculatus* and *N. quadranus* in autumn	1	0.078062	0.078062	0.872997	0.08029	0.571

Effects on variation due season (treat 1 and treat 2), and species (treat 3 and treat 4) are considered. Significant results are marked with an asterisk.

The top five phyla composition of skin microbes in the SpringPn, SummerPn and AutumnPn groups possessed high similarity, although their relative abundance varied (Figure 1; Table 5). In the SummerNq and AutumnNq groups, three of the top five phyla were also identical, but with different relative abundance (Figure 1; Table 5).

**Table 5:** The composition and relative abundance of the top five phyla in each groups.

Phylum	SpringPn	SummerPn	AutumnPn	SummerNq	AutumnNq
Actinobacteriota	33.57% ± 2.33%	1.36 ± 0.28%	7.69 ± 0.68%	1.91% ± 0.35%	9.51% ± 1.46%
Proteobacteria	29.10% ± 3.44%	55.51 ± 10.49%	38.86 ± 2.08%	50.57% ± 5.63%	34.29% ± 2.00%
Firmicutes	16.94% ± 2.05%	26.23 ± 6.44%	6.51 ± 1.01%	29.98% ± 3.68%	\
Bacteroidota	6.10% ± 0.47%	10.52 ± 2.65%	8.85 ± 0.35%	9.79% ± 1.03%	9.12% ± 0.25%
Cyanobacteria	4.79% ± 0.86%	\	\	\	\
Verrucomicrobiota	\	3.28% ± 0.95%	\	3.52% ± 0.81%	\
Acidobacteriota	\	\	8.89 ± 0.64%	\	10.87% ± 0.48%
Gemmatimonadota	\	\	\	\	4.71% ± 0.52%

“\“means the phylum of corresponding group was not appeared in the top five.

**Figure 4:**
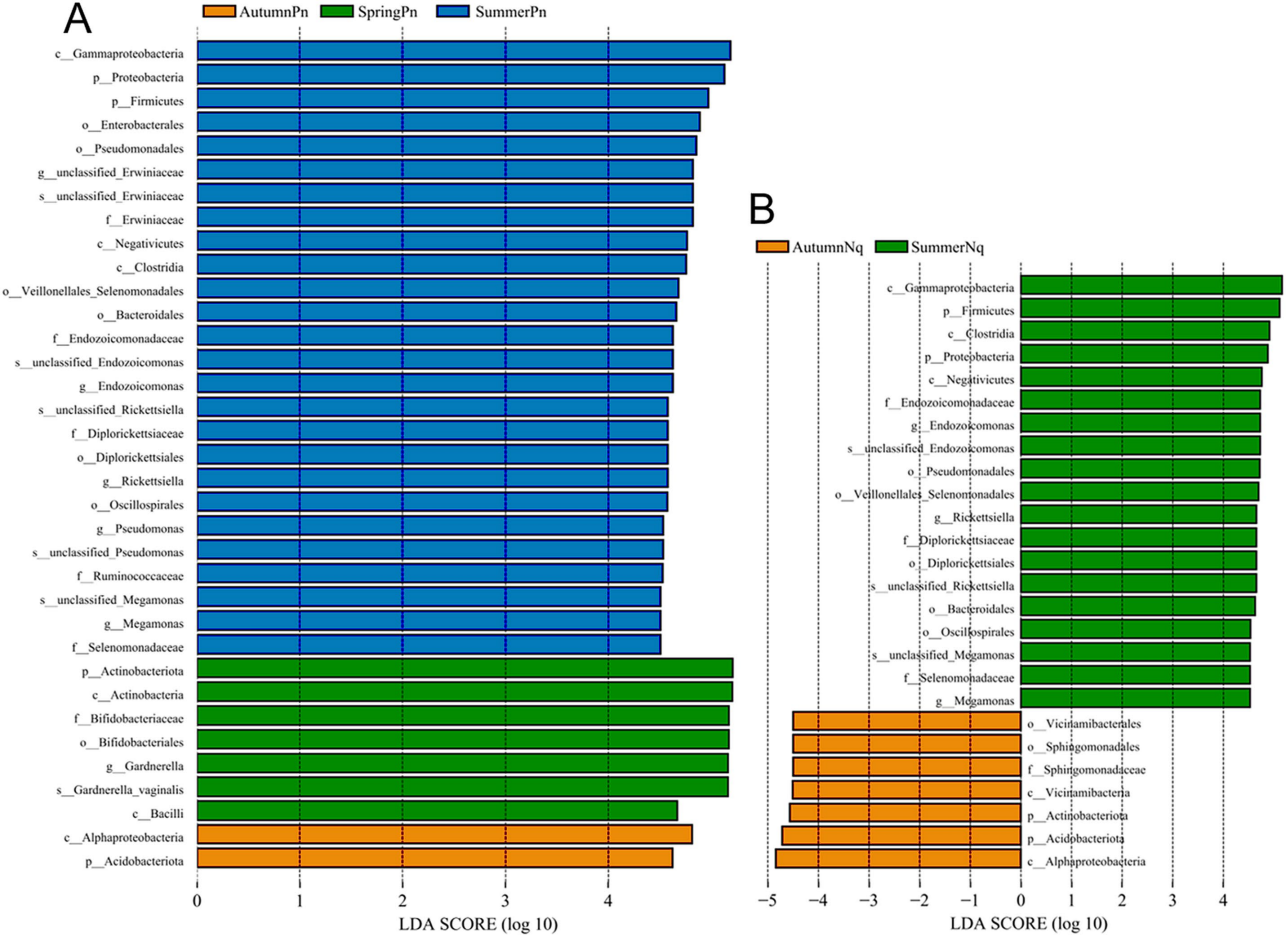
Clustering tree from unweighted pair group method with arithmetic mean (unweighted UniFrac distance) combined with species distribution histogram (genus level) for *P. nigromaculatus* in three seasons (A) and *N. quadranus* in two seasons (B). Clustering tree on the left, species distribution histogram on the right.

The UPGMA clustering tree (unweighted UniFrac distance) combined with the species distribution histogram (genus level) revealed that samples collected in the same season were more similar in species composition (Figure 4). Between-season differences in composition and relative abundance were significant (Figures 4A,B and Table 6). The top five genera observed in AutumnNq accounted for only approximately 20.89% of cutaneous microorganisms (Table 6). The relative abundance of *Lysobacter* (1.97% ± 0.20%) and *unclassified_Microscillaceae* (1.81% ± 0.12%) in AutumnPn group ranked 11th and 12th, respectively, and did not appear in SummerNq group.

**Table 6:** The composition and relative abundance of the top five genera in each groups.

Genus	SpringPn	SummerPn	AutumnPn	SummerNq	AutumnNq
*Gardnerella*	29.28% ± 2.13%	\	\	\	\
*unclassified_Comamonadaceae*	15.41% ± 4.25%	13.17% ± 8.83%	12.08% ± 3.84%	18.15% ± 9.83%	4.15% ± 0.71%
*unclassified_Cyanobacteriales*	4.75% ± 0.86%	\	6.40% ± 1.16%	\	4.66% ± 1.41%
*Ureaplasma*	4.29% ± 0.43%	\	\	\	\
*unclassified_Bacteria*	2.57% ± 0.54%	\	5.38% ± 0.53%	\	4.51% ± 0.31%
*unclassified_Erwiniaceae*	\	13.72% ± 9.90%	\	\	\
*Endozoicomonas*	\	8.12% ± 2.31%	\	10.60% ± 1.99%	\
*Rickettsiella*	\	7.29% ± 1.91%	\	8.88% ± 1.55%	\
*Pseudomonas*	\	7.16% ± 4.54%	\	\	\
*Sphingomonas*	\	\	3.43% ± 0.36%	\	3.92% ± 0.36%
*unclassified_Gemmatimonadaceae*	\	\	2.85% ± 0.33%	\	\
*Megamonas*	\	\	\	6.61% ± 0.97%	\
*Faecalibacterium*	\	\	\	5.06% ± 0.60%	\
*unclassified_Gemmatimonadaceae*	\	\	\	\	3.65% ± 0.36%

“\“means the genus of corresponding group was not appeared in the top five.

**Figure 5:**
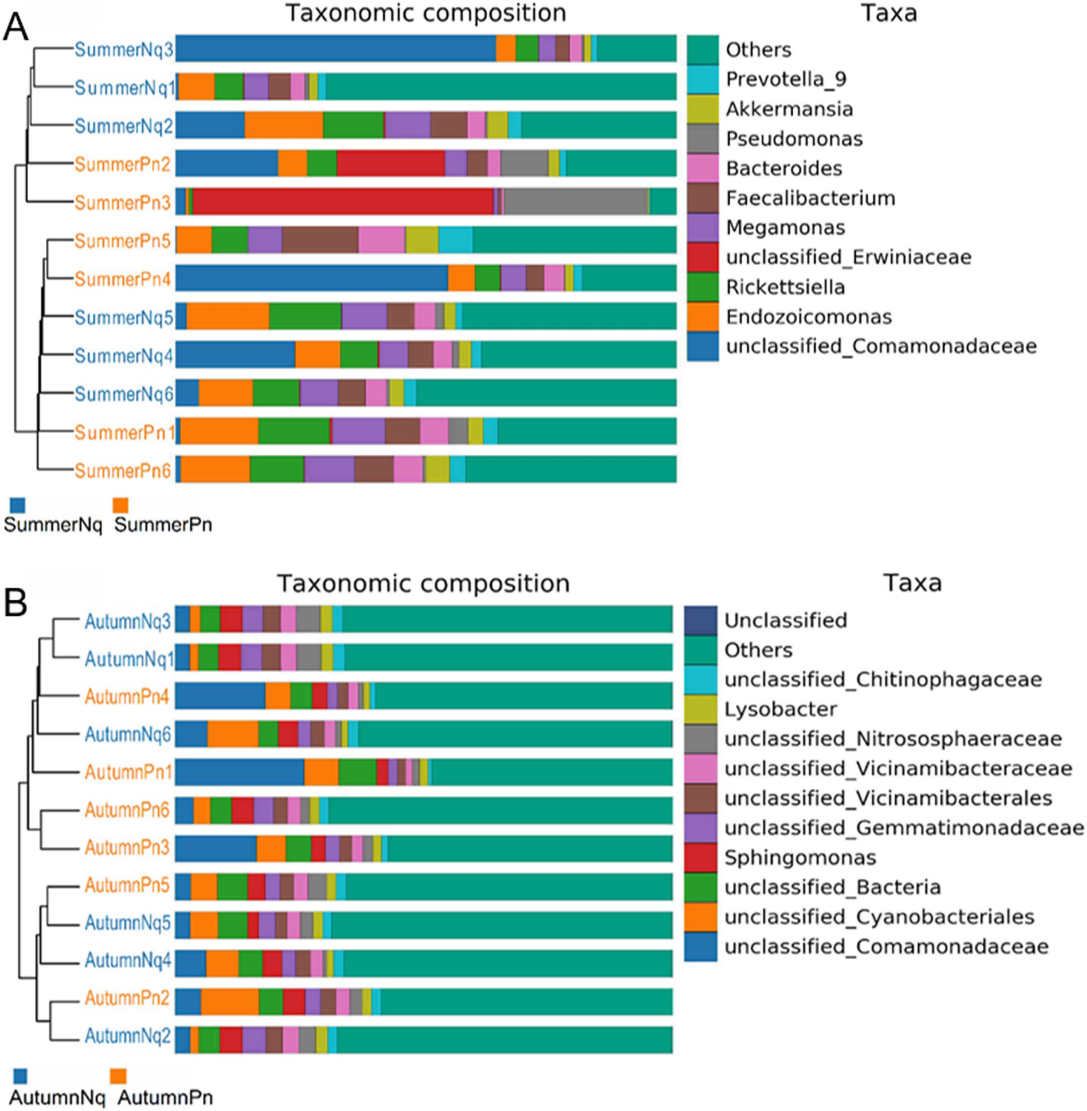
Histogram of the distribution of LDA values (LDA score of 4.5), comparing *P. nigromaculatus* in three seasons (A) and *N. quadranus* in two seasons (B). The vertical axis represents the taxonomic units exhibiting significant differences between the groups, while the horizontal axis displays bar graphs illustrating the logarithmic scores of LDA analysis for each respective taxonomic unit.

Next, LEfSe (LDA score of 4.5) of bacterial populations with between-group differences in relative abundance revealed multiple biomarkers (Figure 5). At the genus level, the most abundant biomarker in SpringPn was *Gardnerella*, while that in summerPn was *unclassified_Erwiniaceae* (Figure 5A). The most abundant biomarkers in SummerNq was Endozoicomonas (Figure 5B).

### 3.3 Effect of host species on cutaneous bacterial communities

The Venn diagram of cutaneous bacterial communities from frog samples revealed that 468 ASVs were shared between SummerPn and SummerNq, accounting for 56.05% of all ASVs in these two groups (Supplementary Figure 4). Additionally, 1,227 ASVs (87.39% of all) were shared between the AutumnPn and AutumnNq groups (Supplementary Figure 5).

**Figure 6:**
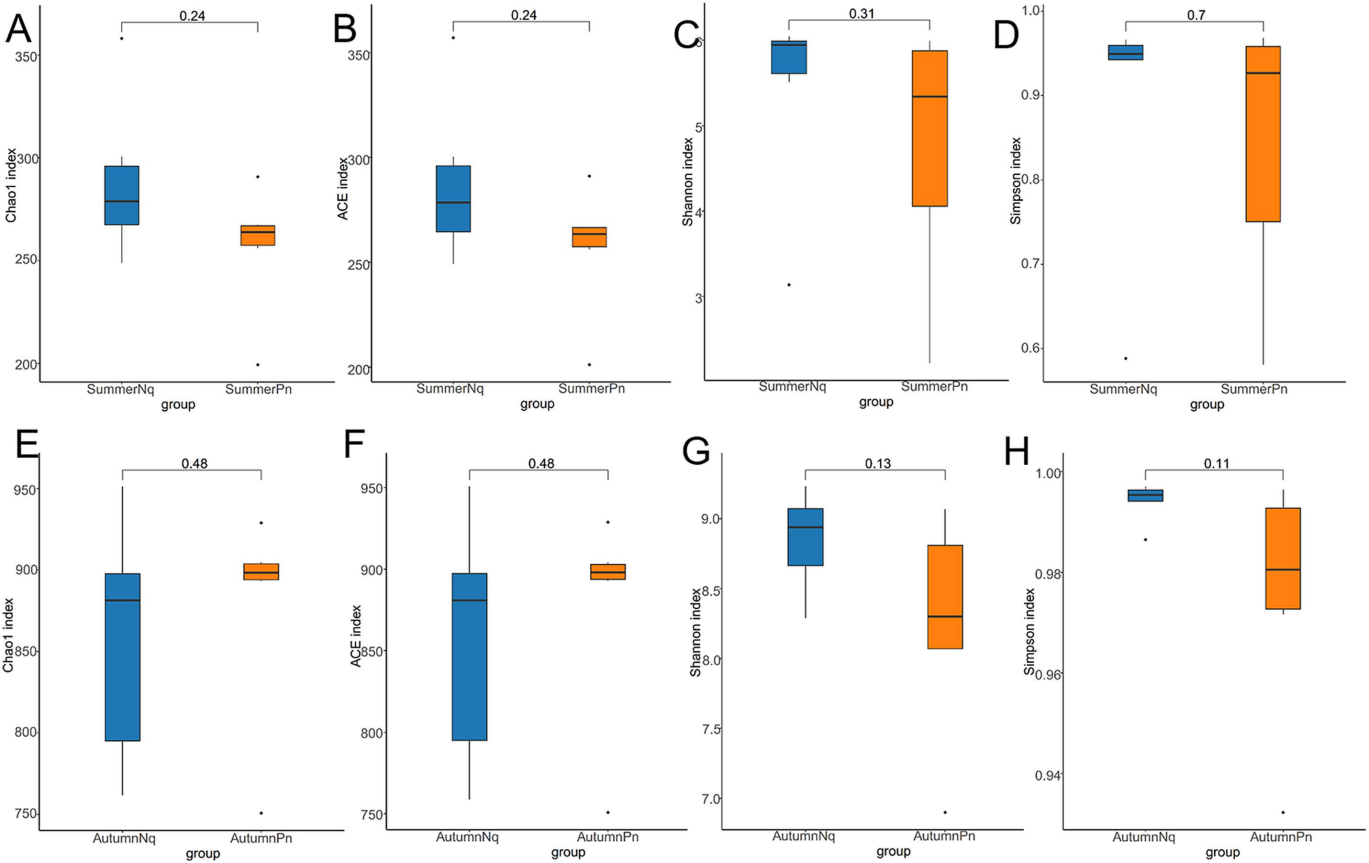
Box plot of variation in alpha diversity indices for cutaneous bacteria on *P. nigromaculatus* and *N. quadranus* in the same seasons, including summer (A–D) and autumn (E–H). The horizontal coordinates are the group names, and the vertical coordinates are the values of the corresponding alpha diversity indices. (A,E) Wilcoxon test of Chao1 index; (B,F) Wilcoxon test of ACE index; (C,G) Wilcoxon test of Shannon index; (D,H) Wilcoxon test of Simpson index.

Alpha diversity did not differ (*p* > 0.05) between the two species during summer or autumn (Figures 6A–H). PCoA analysis demonstrated that the elliptical circles formed by each of the samples of two frog species in same season partially overlapped (Figures 3A,B). Therefore, *P. nigromaculatus* and *N. quadranus* appear to have similar bacterial communities in summer and autumn. The results from PERMANOVA (*p* > 0.05, Treatments 3, 4 in Tables 3, 4) confirmed that the beta diversity of the two frog species did not differ during the same season.

**Figure 7:**
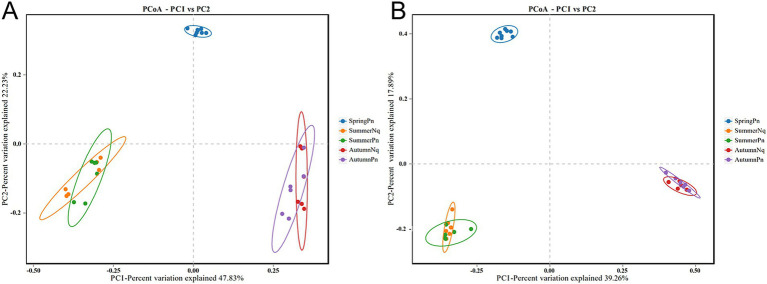
Clustering tree from unweighted pair group method with arithmetic mean (unweighted UniFrac distance) combined with species distribution histogram (genus level) for *P. nigromaculatus* and *N. quadranus* in the same seasons, including summer (A) and autumn (B).

Samples from the two frog species differed in microbial phyla and genera composition and abundance during the same season (Figures 1, 7). The UPGMA clustering tree (Figures 7A,B) indicated that skin samples from the two frog species share similar microbial species composition within the same season.

**Figure 8:**
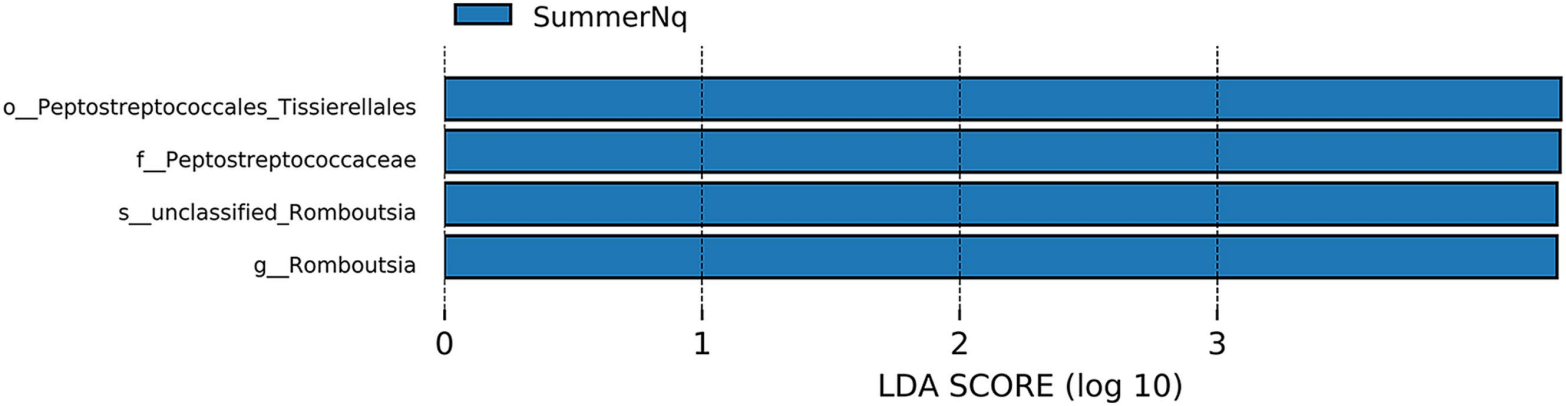
Histogram of the distribution of LDA values (LDA score of 4.0), comparing *P. nigromaculatus* and *N. quadranus* in the same seasons, including summer. The vertical axis represents the taxonomic units exhibiting significant differences between the groups, while the horizontal axis displays bar graphs illustrating the logarithmic scores of LDA analysis for each respective taxonomic unit.

When LDA score was set to 4.0, the SummerNq group had one biomarker, *Romboutsia*, and the SummerPn group had none (Figure 8). Neither frog species exhibited any biomarker in autumn.

### 3.4 Anti-Bd activity was stable across seasonal variation and host species

Of the 1943 ASVs identified, 924 (47.56%) matched the anti-Bd ASV database. Within all samples, anti-Bd ASVs accounted for an average relative abundance of 53.33% and were predominantly from five phyla: Proteobacteria (301), Bacteroidota (164), Firmicutes (161), Actinobacteriota (100), Acidobacteriota (66). Some anti-Bd ASVs appeared only in one frog species, while others appeared in both. We detected 46 anti-Bd bacterial ASVs common to all the groups. These were predominantly Proteobacteria (19), Bacteroidetes (9), Firmicutes (2), Actinobacteriota (5), and Acidobacteriota (6).

The percentage of anti-Bd ASVs varied between groups. The SummerPn group had the highest percentage (327/616, 53.08% anti-Bd ASVs), followed by SummerNq (349/687, 50.80% anti-Bd ASVs), SpringPn (490/1000, 49.00% anti-Bd ASVs), AutumnNq (573/1298, 44.14% anti-Bd ASVs), and AutumnPn (577/1333, 43.29% anti-Bd ASVs). To summarize, in *P. nigromaculatus*, more anti-Bd ASVs were present in the summer than in spring or autumn, with the fewest present in autumn. For *N. quadranus*, more anti-Bd ASVs were present in summer than in autumn.

The variation in the relative abundance of anti-Bd reads (percentage of summed reads associated with anti-Bd ASVs, relative to all reads) differed from that in the percentage of anti-Bd ASVs. The relative abundance of anti-Bd reads in SpringPn, SummerPn, AutumnPn, SummerNq, and AutumnNq was 40.20, 72.56, 46.60, 68.47, and 43.21%, respectively. ANOSIM indicated a significant difference across seasons for *P. nigromaculatus* (Bray-Curtis, R = 0.843, *p* = 0.001) and *N. quadranus* (Bray-Curtis, R = 1, *p* = 0.002). However, anti-Bd reads did not differ across host species in same seasons.

In addition, the PCR results for chytridiomycosis showed all the samples tested negative for Bd and Bsal.

## 4 Discussion

Seasonal fluctuations and host-specific factors can influence pathogen resistance through their effects on the amphibian skin microbiome (Costa et al., 2016; Ellison et al., 2019a; Ellison et al., 2019b; Longo et al., 2015; Muletz-Wolz et al., 2017). Herein, we used samples from *P. nigromaculatus* and *N. quadranus* across different seasons to find empirical support for this hypothesis in anurans from the Qinling Mountains. Our results showed that seasonal variation had a significantly stronger effect on skin bacterial community structure than host species. Stronger effects were observed in changes to microbial alpha-diversity, beta-diversity, species composition and abundance, biomarkers, and anti-Bd function.

Notably, while the phylum level showed higher consistency, the compositions of bacterial communities were much less consistent at the finer taxonomic levels. For example, *Lysobacter* and *unclassified_Microscillaceae* appeared on the skin of both frog species in autumn but was not found in summer and was very rare in spring. *Lysobacter* species are known as “peptide production specialist” and produce peptides that disrupt the cell walls or cell membranes of other microorganisms (Panthee et al., 2016). This means that seasonal variation are associated with peptide production and bacteriostatic action of frog skin. *Gardnerella* was present only in the SpringPn group, despite a high relative abundance. Because we did not capture any *N. quadranus* in spring, we could not verify whether *Gardnerella* spp. would be present on *N. quadranus* in that season. *Gardnerella* is associated with the etiology of human bacterial vaginosis (Shvartsman et al., 2023), but its pathogenic effects on frog skin remain unclear. In addition, *unclassified_Erwiniaceae* appeared in two samples from the SummerPn group. *Erwiniaceae* can produce antibiotics and inhibit the growth of entomopathogenic fungi (Cambronero-Heinrichs et al., 2023). Therefore, the presence of pathogenic fungi in these two samples from the SummerPn group may have caused an increase in *unclassified_Erwiniaceae*. We ruled out the possibility of Bd or Bsal infection in these two samples using PCR.

Our observations on *P. nigromaculatus* and *N. quadranus* suggest that skin bacterial communities are highly dynamic environments. Seasonality in particular had a strong effect on cutaneous bacterial community structure. Bacterial species richness on *P. nigromaculatus* decreased from spring to summer and increased from summer to autumn, while bacterial richness on *N. quadranus* increased from summer to autumn. The consistent increase from summer to autumn may be attributable to new bacterial taxa colonizing the skin. Indeed, we observed that 1,076 ASVs on *P. nigromaculatus* and 1,043 ASVs on *N. quadranus* were present in autumn but not in summer (Supplementary Figures 2, 3). Notably, *P. nigromaculatus* and *N. quadranus* showed similar variation in community structure despite being different species and from different locations. In both frogs, microbial species richness and diversity were significantly greater in autumn than in summer. Additionally, Actinobacteria was more abundant in autumn than in summer, whereas Proteobacteria was more abundant in summer than in autumn.

The prominent seasonal shift in bacterial community structure in these two aquatic frog species is probably related to changes in water temperature at both locations. Temperature directly influences the growth of bacterial community assemblages, community-member interactions, and antifungal functions (Bletz et al., 2017b; Daskin et al., 2014; Woodhams et al., 2014). Although environmental microorganisms were not sampled and analysed in this study literature shows that environmental temperature can alter amphibian gut microbiota and microbial communities in surrounding habitats (Bletz et al., 2017b; Kohl and Yahn, 2016). For aquatic frogs, bacteria in paddy fields, ponds, rivers, and near-water soils are important microbial reservoirs that colonize the skin; bacterial communities in these habitats also exhibit considerable temporal variation (Crump and Hobbie, 2005; Rasche et al., 2011). Therefore, seasonal (temperature-related) shifts in bacterial communities may dictate host-associated microbiota variations. The absence of environmental controls lead us to the current lack of clarity on the changes in the bacterial community specific to frog skin.

Anti-Bd bacterial ASVs (47.56%) within our all samples were higher than those reported on anuran species from Panama (8.47%), but lower than those reported from India (51.7%) (Mutnale et al., 2021; Varela et al., 2018). The factors contributing to the varying percentages of anti-Bd ASVs included soil pH, precipitation, prevalence of Bd infection, and others (Mutnale et al., 2021; Varela et al., 2018). Notably, the average percentage of anti-Bd ASVs in summer (51.94%) were higher than those in spring and autumn in our study. Therefore, the sampling season had a significant effect on the percentage of anti-Bd bacterial ASVs.

The top five most abundant genera in SummerPn all possessed anti-Bd properties, representing 60.15% of anti-Bd ASV abundance in the group. Overall, *P. nigromaculatus* skin bacteria in summer had the highest anti-Bd ASV abundance (72.56% average relative abundance), but all the test groups exhibited potential anti-Bd activity. This functional stability resulted from the constant presence of specific ASVs and replacement by different ASVs in some seasons. For example, anti-Bd *Pseudomonas* ASV (ASV 376) and *Acinetobacter* ASV (ASV 1770) were consistently present across all the three seasons, whereas anti-Bd *Serratia* ASV (ASV 162) only appeared in spring for *P. nigromaculatus* and autumn for *N. quadranus*. Moreover, *Janthinobacterium* ASV (ASV 31908) only appeared in spring and summer for *P. nigromaculatus*. *In vitro* experiments have demonstrated that *Pseudomonas* and *Acinetobacter*, isolated from amphibians, produced metabolites with Bd inhibitory activity (Brucker et al., 2008b; Becker et al., 2015; Woodhams et al., 2015). Additionally, *Janthinobacterium lividum* and *Serratia marcescens* can produce anti-Bd (and generally antifungal/antibacterial) metabolites such as prodigiosin and violacein (Woodhams et al., 2018; Zhang et al., 2024). Therefore, our predicted anti-Bd genera align with prior finding from *in vitro* experiments. Overall, seasonal shifts in host-associated bacterial communities were associated with significant variation in potential anti-Bd functions. Consequently, hosts may temporarily lose important protective bacterial taxa during certain seasons, drastically increasing their susceptibility to this disease.

Anti-Bd bacteria can produce Bd inhibitory metabolites or biofilms on frog skin. The host itself may also possess mechanisms to select anti-Bd metabolite-producing bacteria on the skin (Woodhams et al., 2018; Mutnale et al., 2021; Loudon et al., 2014; Piovia-Scott et al., 2017; Loudon et al., 2016). In this study, we demonstrated that the two tested frog species exhibited abundant anti-Bd bacterial ASVs on the skin. However, it is unclear whether this is directly related to the temporary absence of Bd infections in anuran populations from the Qinling Mountains. Moreover, the anti-Bd ASV database assembled by Woodhams et al. (2015) does not include any sampling from Qinling Mountains of China; hence, it cannot be ruled out that there are certain bacteria in the Qinling Mountains that might have anti-Bd activity but have not been matched with the anti-Bd ASV database. This area may be the focus of our subsequent studies.

## 5 Conclusion

Our results indicated that seasonal variation exerted a greater effect than host species on the structure and anti-Bd function of cutaneous bacterial communities. These communities exhibited more consistent structural similarities at the phylum level but were more diverse at finer taxonomic levels. Although both frog species hosted bacteria with anti-Bd function, the skin of *P. nigromaculatus* during summer had the highest anti-Bd bacterial ASV abundance. Our study provides important insights into the seasonality of cutaneous bacterial community structure on amphibians inhabiting the Qinling Mountains. The findings establish foundational knowledge for future studies on host–bacteria interactions and the structure–function relationship within amphibian skin bacterial communities. The findings from this study and other related studies can potentially benefit research on disease etiology and control in other wildlife hosts of cutaneous microbes.

## Data Availability

The datasets presented in this study can be found in online repositories. The names of the repository/repositories and accession number(s) can be found below: https://www.ncbi.nlm.nih.gov/, NCBI BioProject, PRJNA1134887.

## Footnotes

^1^ http://www.biocloud.net/

^2^ http://cloudtutu.com.cn/
