# Supplementary material for: Seasonality influences skin bacterial community structure and anti-Bd function in two anuran species
Source: Front Microbiol. 2024 Nov 5;15:1463563. doi: 10.3389/fmicb.2024.1463563 (PMC11573762; doi:10.3389/fmicb.2024.1463563)
Supplement: Supplementary file 1 [file Data_Sheet_1.docx]

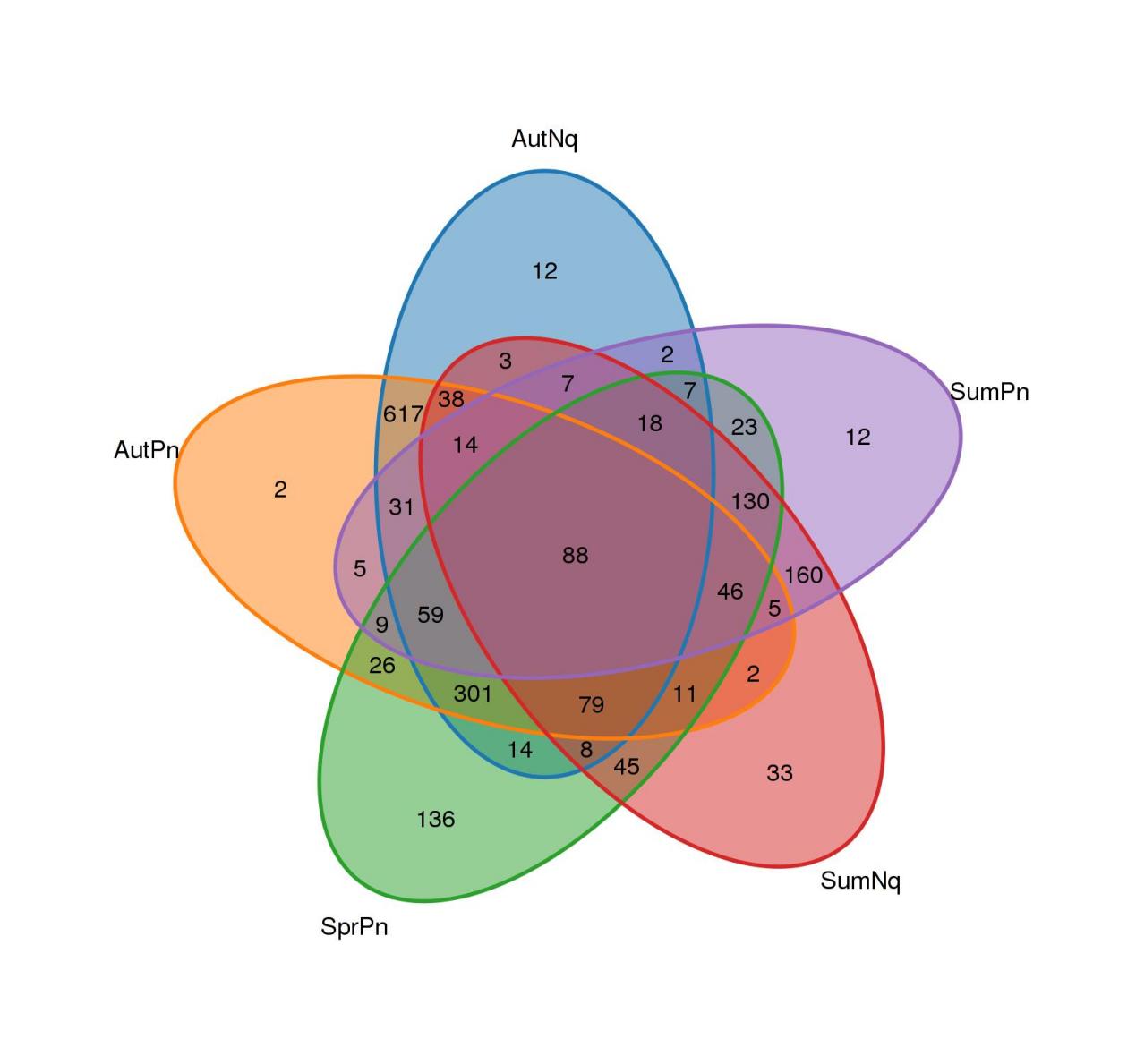


**Supplementary Figure 1** Venn diagram presenting number of shared, unique ASVs between each groups of all samples.


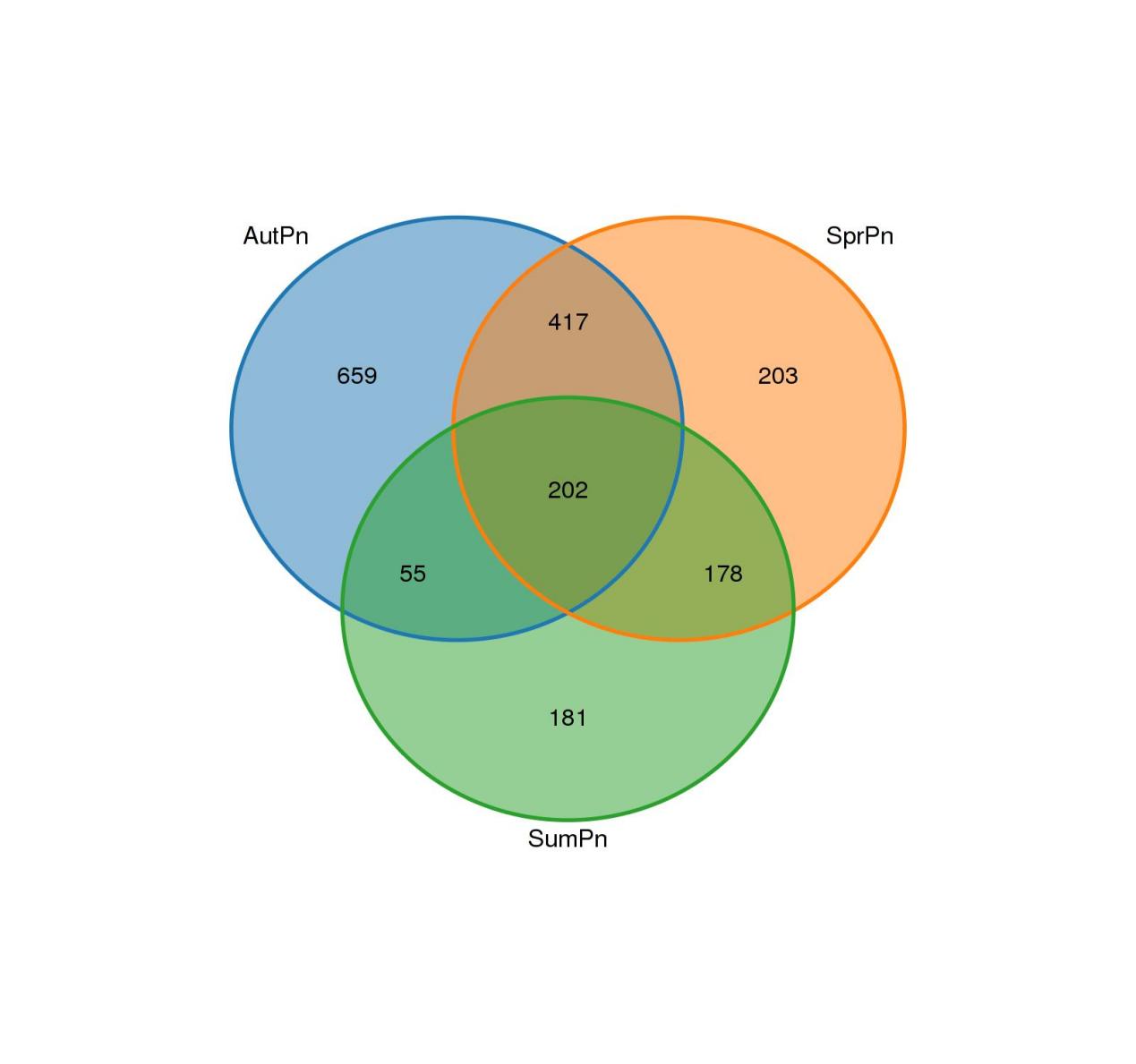


**Supplementary Figure 2** Venn diagram presenting number of shared, unique ASVs between each groups of *Pelophylax nigromaculatus*.


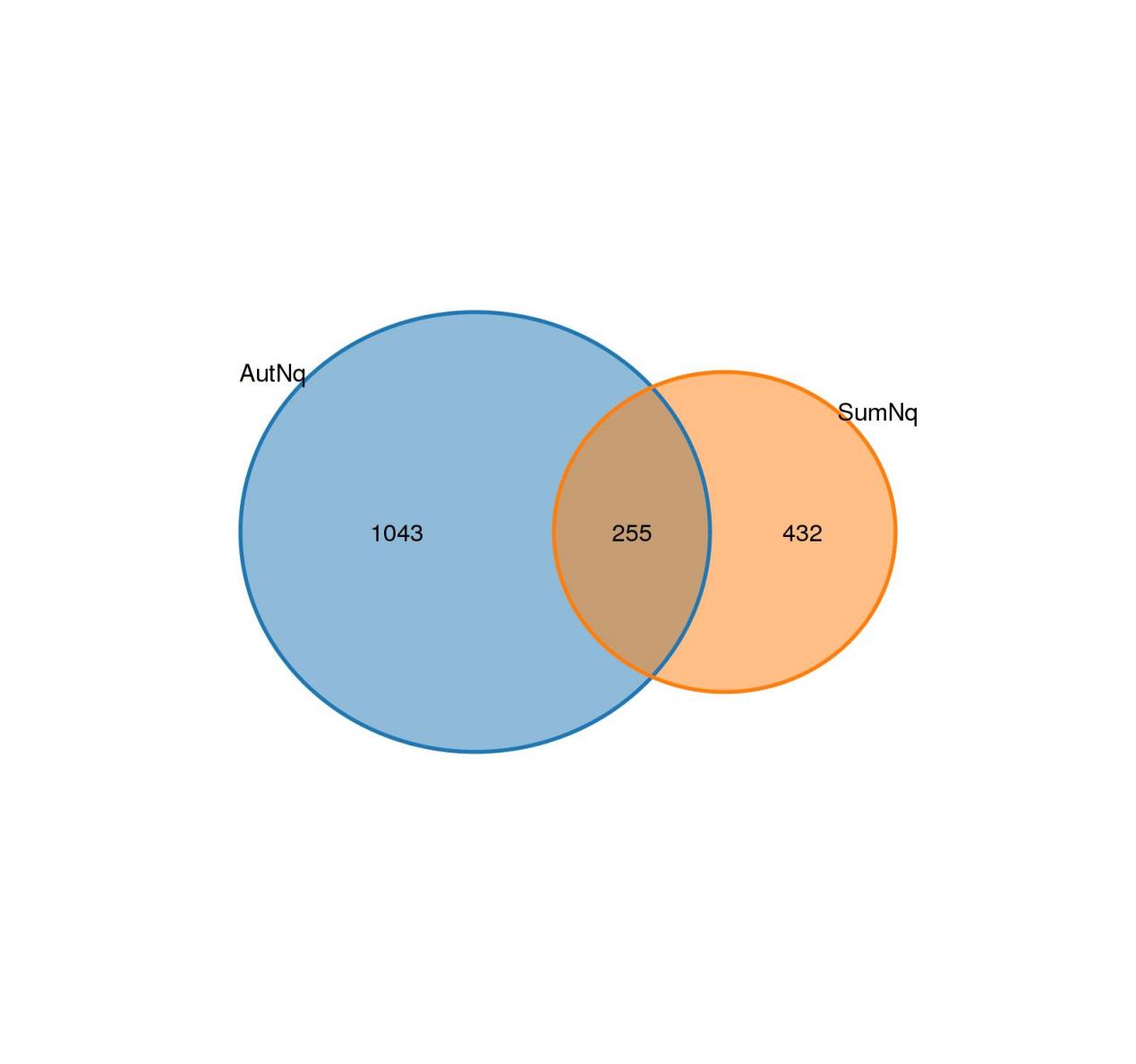


**Supplementary Figure 3** Venn diagram presenting number of shared, unique ASVs between each groups of *Nanorana quadranus*.


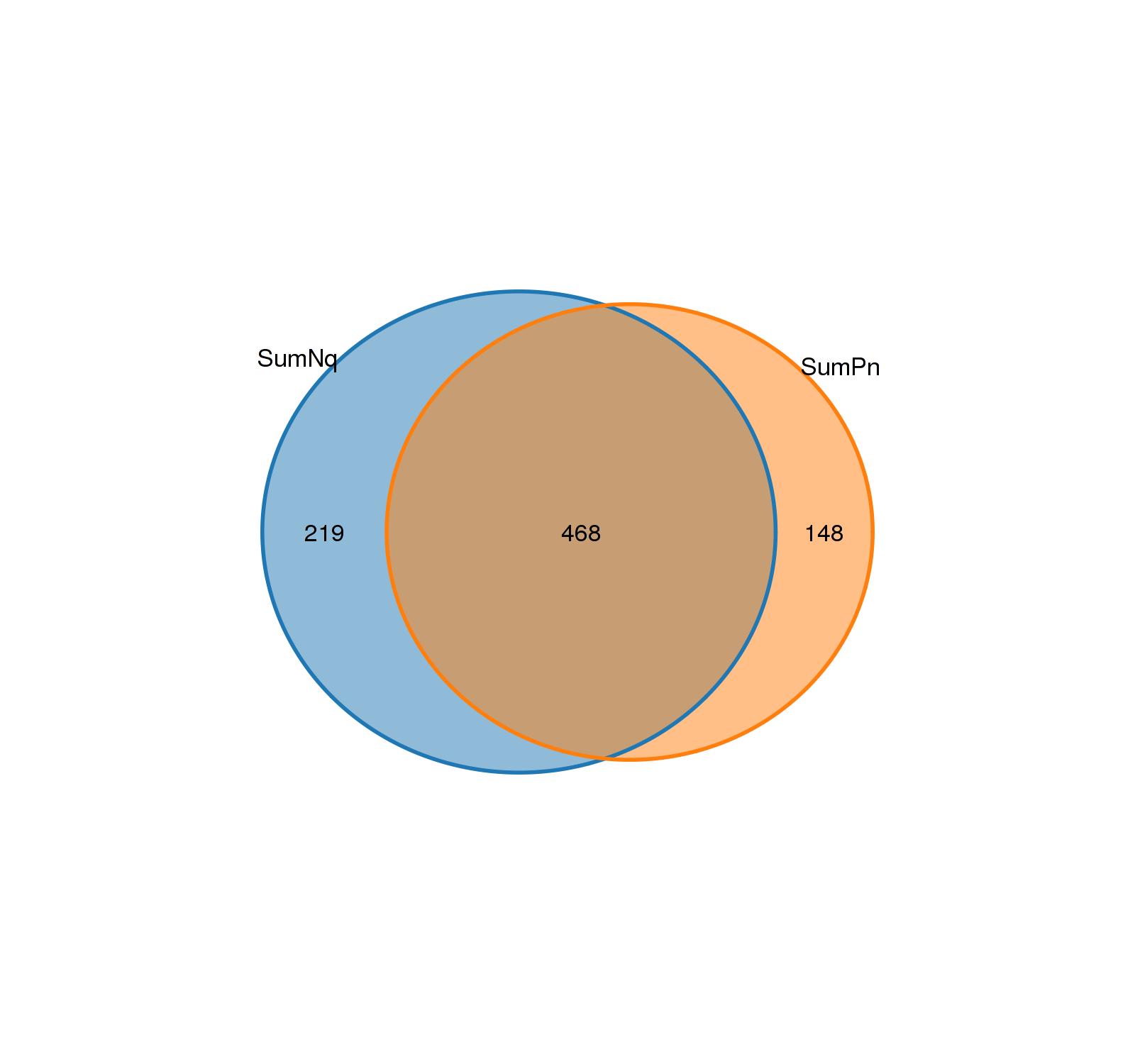


**Supplementary Figure 4** Venn diagram presenting number of shared, unique ASVs between *P. nigromaculatus* and *N. quadranus* in summer.


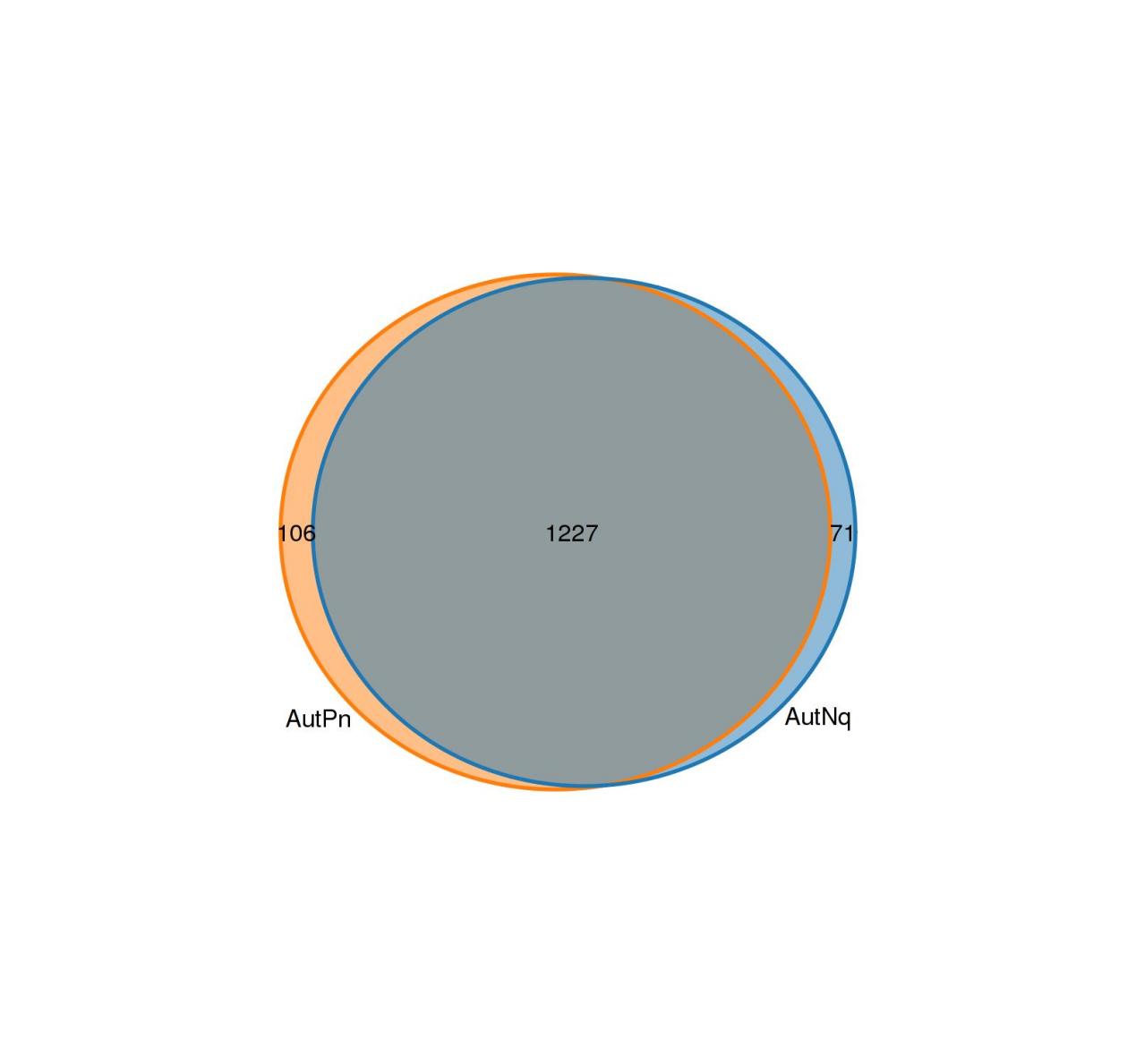


**Supplementary Figure 5** Venn diagram presenting number of shared, unique ASVs between *P. nigromaculatus* and *N. quadranus* in autumn.
